# Technical Note: Pedicle Cement Augmentation with Proximal Screw Toggle and Loosening

**DOI:** 10.1111/os.12467

**Published:** 2019-06-09

**Authors:** Wen Jie Choy, William R Walsh, Kevin Phan, Ralph J Mobbs

**Affiliations:** ^1^ NeuroSpine Surgery Research Group (NSURG) Sydney New South Wales Australia; ^2^ Faculty of Medicine, University of New South Wales Sydney New South Wales Australia; ^3^ Surgical & Orthopaedic Research Laboratory, Prince of Wales Clinical School University of New South Wales, Sydney Randwick New South Wales Australia; ^4^ Prince of Wales Private Hospital New South Wales Australia

**Keywords:** Cement augmentation, Fenestrated pedicle screws, Lumbar spine, Peri‐screw halo, Screw loosening, Screw toggling

## Abstract

**Background:**

Cement augmentation is a technique used to increase the stability and purchase of pedicle screws in poor quality bone. Various methods can be applied for cement delivery, such as cement injection before screw placement and the use of fenestrated screws. However, potential problems can arise with the use of cement augmentation.

**Case Presentation:**

A 66‐year‐old man with a lower trunk deformity, severe kyphosis, and sagittal imbalance following fusion (L_2‐5_), with minimal comorbidities, was referred to our unit 9 months after surgery. Pain and progressive kyphosis were investigated clinically and radiographically with computed tomography (CT) scans to assess the status of the hardware and fusion. CT imaging revealed that cement was present only at the distal tip of the fenestrated screws at the L4 vertebral level. A non‐union was present along with loosening and a halo around the body of the pedicle screws, and there was evidence of pullout of inferior screws.

**Conclusion:**

Single‐level cement augmentation of pedicle screw in a posterior construct and distal tip cement augmentation of the screw results in a fixed pivot point. Micromotion in cranio‐caudal loading during flexion and extension may result in screw toggling with the single‐level cement‐augmented tip as a fulcrum. This may cause screw loosening, which can lead to pullout and loss of construct stability. The halo around the screw suggests bone loss and/or a fibrous tissue interface, which further complicates revision surgery. Stress shielding and polymethylmethacrylate cement present additional difficulties.

The findings of this technical note question the risks and benefits of cement‐augmented fenestrated pedicle screw fixation for spinal fusion. Although incidences of such cases are uncommon, surgeons should perform this technique with caution. Accurate restoration of lumbar lordosis during index procedures is important to minimize the risk of construct failure.

## Introduction

**Figure 1:**
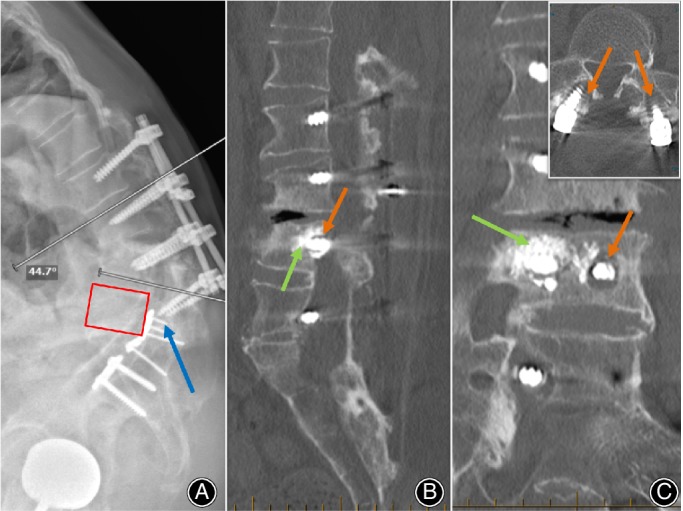
(A) Standing full‐body EOS scan; red box showing position of L_5_ vertebral body; blue arrow showing pull‐out of L_5_ pedicle screws. The acute kyphotic angle at L_3/4_ due to the construct failure was approximately 45^o^. (B) Sagittal CT with L_2_‐L_5_ pedicle screw construct with L_4_ cement‐augmented pedicle screws in vertebral body (green arrow). (C) L_4_ cement‐augmented pedicle screws (green arrow) on coronal CT view. Inset: L_5_ Proximal screw toggle with halo around pedicle screw and loosening of construct. Orange arrows indicate peri‐screw haloing due to cranial‐caudal toggling.

The use of vertebral screws for spinal fixation dates back to the 1940s and has since evolved into pedicle screw fixation for stabilization to encourage intervertebral arthrodesis^1^ and for the treatment of various spinal pathologies such as stabilizing traumatic injuries, correction of deformities and spinal fusion. Various methods of pedicle screw fixation have been used including minimally invasive percutaneous screw fixation, open pedicle screw insertion^2^, ^3^, ^4^, and more recent trajectories including the cortical screw have gained popularity^5^, ^6^. To achieve improved stability and purchase, vertebral body and pedicle screw augmentation (kyphoplasty via the central screw cannulation and distal holes, and transpedicle injection) utilizing different bone cements such as calcium apatite cement (CAC), injectable calcium phosphate (CaP) and polymethylmethacrylate (PMMA) have been trialled^7^, ^8^, ^9^, ^10^. Multiple techniques of cement delivery are currently in practice. Formerly, bone cement was injected into the drilled lumen before insertion of the pedicle screw; recently, fenestrated pedicle screws (Fig. 1B) have been utilised which allows cement, such as PMMA, to be injected through the screw into the vertebral body after the screw is in place^11^, ^12^, ^13^, ^14^. In the setting of decreased bone mineral density (BMD) such as osteoporosis, cement augmentation is proven to improve pedicle screw purchase and outcomes^15^, ^16^.

However, potential problems can arise with cement augmentation. Intraoperative mis‐positioning of screw holes^17^ and cement leakage;^18^ cement leakage into surrounding structures and post‐operative screw migration;^19^ which all can result in significant neurological deficit, morbidities or mortality such as pulmonary embolism^14^, ^18^, ^19^, ^20^, ^21^. Incomplete curing of PMMA upon injection can also cause thermonecrosis of surrounding neural structures^22^. Moreover, micromovements, loading, and twisting/ rotational movements of the relevant spinal motion segments before complete osseointegration of the fusion construct can predispose to non‐union.

Although clinical studies discussing the potential toggling of pedicle screws have been carried out, to the authors’ knowledge there is no formal publication reporting the outcomes of screw toggling in patients. This technical note will discuss the outcomes of distal cement augmented pedicle screw which serves as a pivot point for proximal screw toggling and loosening.

## Case Presentation

We report a case of a 66‐year‐old male with minimal co‐morbidities who presents with lower trunk flat back deformity, severe iatrogenic kyphosis and sagittal imbalance following 9 months postoperative lumbar decompression and fusion (L_2_‐L_5_) with postero‐lateral grafting, without inter‐body implants from a different institute. He experienced significant pain due to L_4_ and L_5_ motor / sensory radiculopathy. Reflexes were absent in both lower limbs with a bilateral foot drop. Oswestry Disability Index was 74%, with Visual Analog Scale pain score of 10 in the standing position, 8 on lying flat.

**Figure 2:**
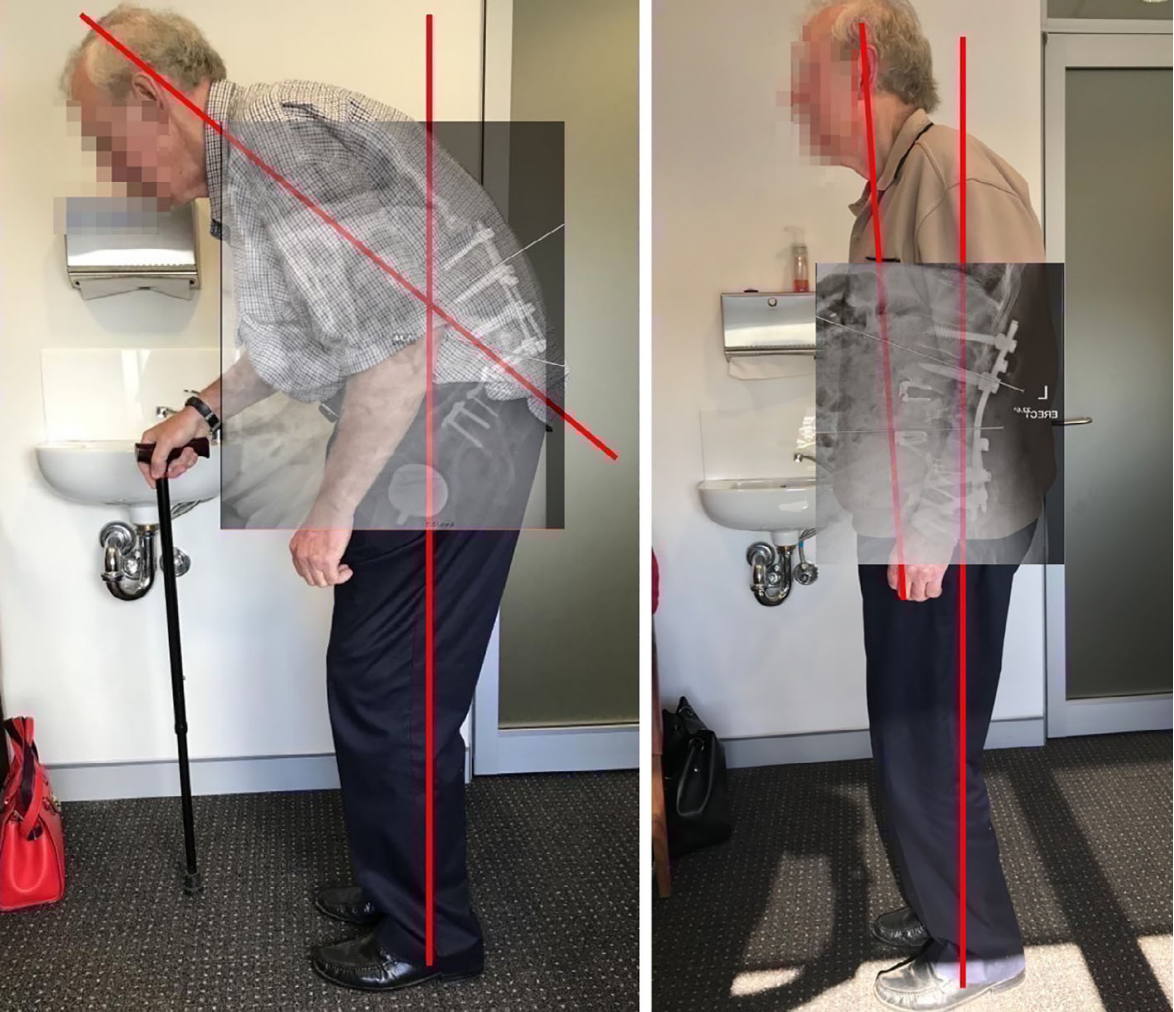
(A) Pre‐operative image of patient with gross sagittal deformity and bent‐knees to accommodate for the kyphosis in the lumbar spine in order to maintain a gaze along the horizon. (B) Post‐operative image of patient with a corrected sagittal posture and requiring no walking aid for ambulatory.

Computed tomography (CT) scan revealed loosening and non‐union of the inferior aspect of the fusion construct. There is pull‐out of the inferior screws (Fig. 1A) with haloing around the body and distal aspect of the pedicle screws. (Fig. 1). Standing EOS scan reveals gross sagittal imbalance. The kyphotic angle due to the construct failure at L_3/4_ level was approximately 45°. A flat back deformity can be seen along the vertebral levels above the level of screw pull‐out. Bone mineral density was normal for his age. Figure 2A shows the pre‐operative presentation, with significant sagittal plane deformity and bent‐knees in order to maintain a gaze along the horizon while requiring a walking aid for ambulatory.

The haloing effect demonstrated by the pedicle screws is consistent with hardware failure, and also seen in the “PEEK‐Halo” effect when PolyEther‐Ether‐Ketone (PEEK) is being used as an intervertebral implant which results in poor osseointegration^23^. However, in this case, the poor osseointegration was a result of repetitive screw cranial‐caudal micromotions and toggling which prevent consistent screw – bone contact but not due to hardware material.

**Figure 3:**
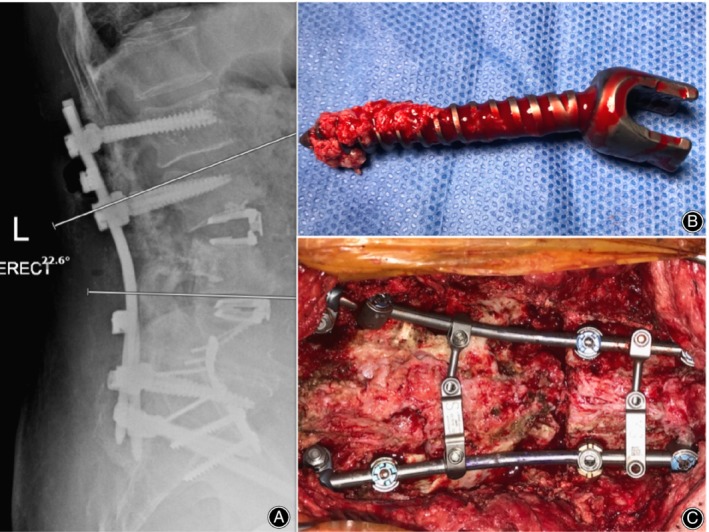
(A) Post‐operative standing X‐Ray of patient. Lordosis was restored utilizing 2 ALIF cages in L_3/4_ and L_4/5_ level post‐removal of previous construct. Revision construct with up‐sized L_2_ and L_3_ pedicle screws combined with S_1_ and S_2_AI screws were used to provide a distal construct foundation. A focal lordotic correction at L_3/4_ of approximately 22^o^ was restored. (B) Removed right L_4_ cement‐augmented pedicle screw with cement surrounding the screw tip. (C) Revision with Anterior construct (ALIF) and Posterior Smith‐Peterson osteotomy to restore lordosis of the lumbar spine.

A revision procedure utilizing both anterior and posterior approach was carried out (Fig. 3). Lordosis was restored utilizing 2 anterior lumbar interbody fusion (ALIF) cages (L_3/4_ and L_4/5_ levels) and Posterior Smith‐Peterson osteotomy at L_3/4_. Focal lordosis of approximately 22^o^ was corrected resulting in a total correction of over 65 degrees at L_3/4_. The patient was able to stand up‐right which enabled him to maintain his view on horizon when his spine was in a neutral position. Figure 2B shows patient post‐op with a corrected posture requiring no walking aids to ambulate.

## Discussion

To the authors’ knowledge, there is currently no literature reporting such an adverse event, however the use of fenestrated cement fixation is relatively recent. A cadaveric study by Bostelmann et al. studying the effect of cranio‐caudal cyclic loading on non‐augmented and cement‐augmented pedicle screws concluded the superiority of augmentation compared to standard pedicle screws^24^. A separate study comparing sequence of cement augmentation before or after reduction manoeuvre of pedicle screws in a cadaveric study showed the superiority of cement augmentation post‐reduction to reinforce screw purchase^25^. However, the end effect of toggling due to cranio‐caudal loading was not studied.

**Figure 4:**
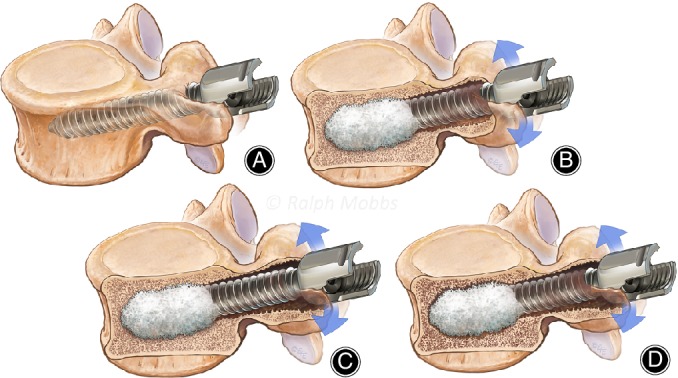
Failure of Distal Cement Augmentation of Pedicle Screw. (A) Pedicle screw position with distal fenestration for cement augmentation. (B) Cement delivery via distal fenestration. Non‐union may lead to toggle of screw and proximal loosening and failure. (C) Further movement will increase halo effect around proximal screw insertion point. (D) Halo and fracture of cement with pull‐out of screw.

In the case of fenestrated screws were only the tip is strongly fixated by cement in the vertebral body, the tip can act as a fulcrum in which micro‐movements and torsion can cause toggling of the screw body within the pedicle, resulting in proximal loosening, and non‐union of the fusion construct. As time passes, cranio‐caudal loading during lumbar flexion and extension, accumulates into a significant amount of force which eventually results in minute motions turning into significant toggling upon the cemented fulcrum. Further loading result in more proximal toggling. The loss of bone structure around the screw appears as a haloing effect seen on imaging (Fig. 4). The toggling effect can be further exacerbated if there is loss of bone mineral density which allows the screws to pivot easily with movements and torsion of the spine. These motions can also result in loss of cortical bone which weaken the screw fixations^26^. Eventually, this can lead to proximal and distal screw loosening and affect the cemented region as in this case.

The reduced forces withstood by the spine may be a potential risk factor for stress shielding to occur. As seen in the hip, stress reduction in an implanted bone can lead to bone loss^27^. Weight‐bearing is an important factor that promotes bone growth and deposition^28^. Inter‐body constructs serve as a secondary anterior column support for the spine^29^ and may result in reduced load being transferred through the pedicle screw construct, as in the current case. With a patient fixated with poor sagittal balance and no anterior column support, coupled with toggling and micro‐movements of the posterior pedicel screw, the chances of non‐union further increases.

The end result of such an effect can be disastrous to the patient. Revision of the loose pedicle screws presents a significant problem, as removal of these screws may lead to further damage of the surrounding bone or fracture of the vertebral body and the pedicles. The patient may require further levels of fusion as well as other invasive surgeries (e.g. anterior or lateral approaches) to correct the kyphosis. Blattert et al. argued that removal of cement‐augmented fenestrated screws was possible even in severe osteoporosis. They concluded that the cement screw connection was fragile enough to break off during extraction^30^. However, Bullmann et al. showed the axial pull‐out strength and torque is significantly higher in cement‐augmented fenestrated screws compared to non‐augmented screws. These were associated with pedicle and vertebral body fractures. Additionally, there is an increased chance of cement leakage if cement augmentation is used during revision^31^. Moreover, there are limitations associated with in‐vitro lab studies. Osseointegration and bone remodelling is not applicable in such settings as cadaveric bones do not heal. The end result of bone remodelling may affect the stability of the construct with time.

Different methods have been proposed and used for the revision of non‐augmented pedicle screws. These include the use of a larger and longer pedicle screws^32^, cement augmentation of the revised screws^33^ and pre‐operative planning of different trajectory for revision screw placements^34^. However, there are currently no promising techniques for the revision of cement‐augmented fenestrated screws. Removal of the screws may risk fracturing the vertebrae and neurological injury with screw/cement removal. Mesfin et al. reported a revision for a failed cement‐augmented fenestration screws fixation in a osteoporotic patient by extending the posterior construct, cement‐augmentation, and addition of a titanium hook and woven polyester band to increase the points of fixation^35^. If, however, the cement was broken off from the screws during revision as described by Blattert et al.,^30^ the residual cement can potentially act as a barrier for proper osseointegration between bone and the newly inserted screw. A cement‐in‐cement revision technique which is commonly used in hip arthroplasty^36^, ^37^ may be required to aid stability for the revised construct.

In addition to extending the posterior construct for re‐establishing sagittal balance, the authors agree that the use of interbody fusion techniques may further benefit the patient for anterior column support. In terms of correcting sagittal alignment, the use of ALIF as in the current case has been shown to compliment other realignment techniques^38^, ^39^. A minimally invasive, lateral transpsoas approach which incorporates lateral interbody fusion and anterior longitudinal ligament release reported by Murray et al. and Pimenta et al.^40^, ^41^ is another promising technique in this scenario.

Further studies and data are required to study the long‐term efficacy of cement‐augmented pedicle screw fixation, associated risks and complications. Long‐term outcomes of pedicle screw fixation comparing different screws should be carried out to compare the pros and cons of different screw designs. Recently, expandable pedicle screws have gained an interest amongst spine surgeons^42^, ^43^. Standalone comparison of expandable pedicle screws with standard pedicle screws demonstrated the former has a greater peak pullout force^44^. Aycan et al. suggested due to toggling and the brittleness of PMMA, expandable pedicle screws with PEEK shell might be a better alternative in dynamic loading^45^. Nevertheless, major complications such as the one reported here will further question the safety and efficacy of this technique. Better alternatives should be sought out to reduce the chance of such adverse outcomes in addition to a surgical focus on alignment correction with the initial surgery^46^.

### *Conclusion*

The use of single‐level cement augmentation has resulted in a fulcrum for screw toggling when load was applied along the construct. Accurate restoration of sagittal alignment with ideal lumbar lordosis at the index procedure while using cement augmentation techniques would have reduced the risk of such a complication. Separately, the use of cement‐augmented fenestrated pedicle screws is a recent technique. Although there is currently an adequate body of literature on the pull‐out strength and stability of cement‐augmented fenestrated pedicle screws, there are no reports on non‐union biomechanics and the effect of cranio‐caudal toggling. Haloing around pedicle screw due to toggling is a major complication especially if cement‐augmentation was used. This phenomenon adds significant difficulty and potential morbidity to the revision procedure. Anterior or lateral interbody cages may aid with sagittal realignment. The appearance of such phenomenon potentially questions the role of such fixation technique. The use of intra‐operative on table CT scan may be beneficial to access the accuracy of screw placement in order to limit post‐operative toggling due to abnormal stress loading.

## Declaration

All authors contributed significantly and met the authorship criteria according to the latest guidelines of the International Committee of Medical Journal Editors. All authors agree to the final submitted manuscript.
